# Supplementary figures and images for: B cell expression of an enzymatic intermediary in ether lipid biosynthesis promotes antibody responses and germinal center size
Source: eLife. 2026 Apr 27;14:RP104580. doi: 10.7554/eLife.104580 (PMC13120822; doi:10.7554/eLife.104580)

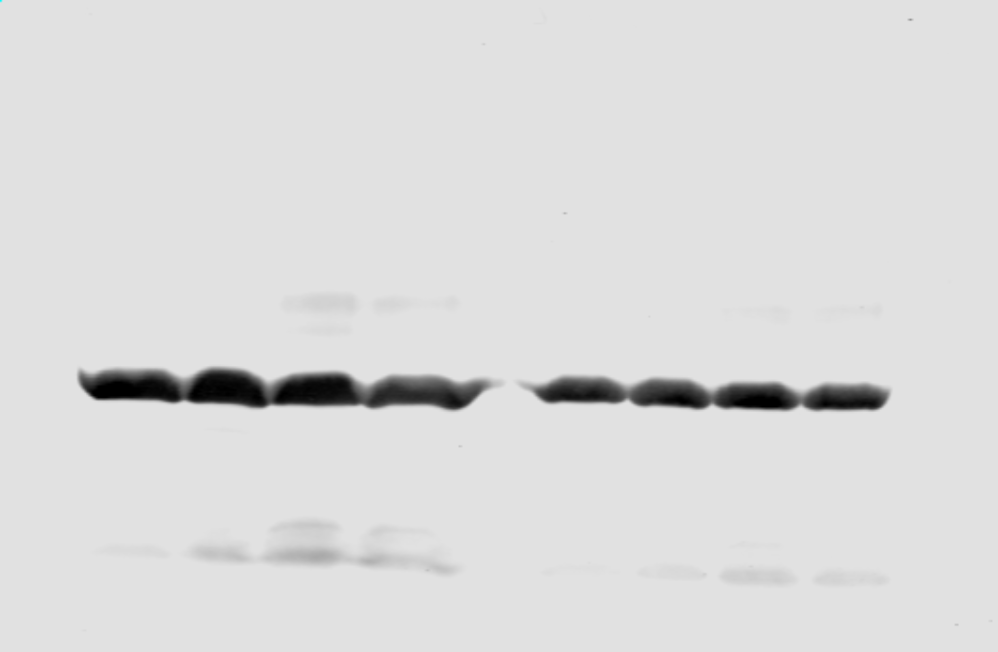

Supplement: Figure 1—source data 1. [file elife-104580-fig1-data1.zip › Figure 1B_Source Data-1 /PexRAP cKO_hCD20-CreER_B T_ Actin WB.tif]

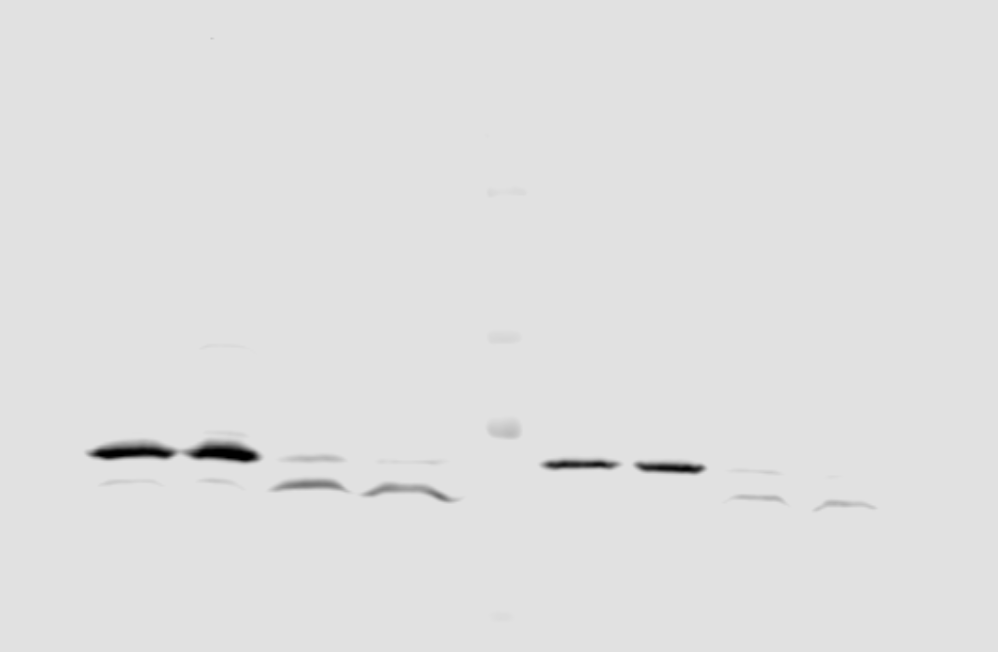

Supplement: Figure 1—source data 1. [file elife-104580-fig1-data1.zip › Figure 1B_Source Data-1 /PexRAP cKO_hCD20-CreER_B T_ PexRAP WB.tif]

Total cell lysate:      120 µg      40 µg

| WT |   | <i>Dhrs7b</i> <sup>Δ/Δ</sup> |   | WT |   | <i>Dhrs7b</i> <sup>Δ/Δ</sup> |   |
|----|---|------------------------------|---|----|---|------------------------------|---|
| B  | T | B                            | T | B  | T | B                            | T |

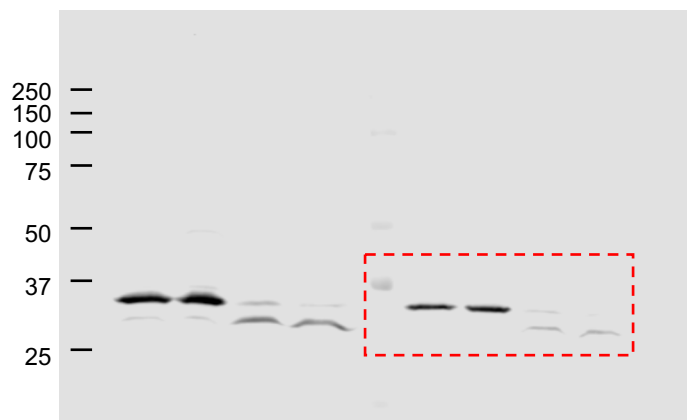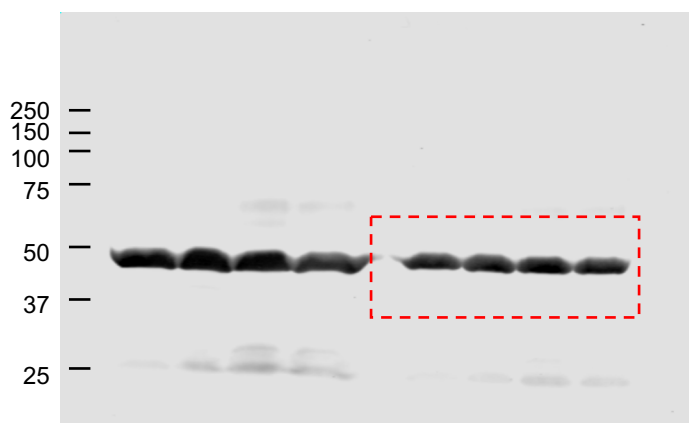

Figure 1B-Source Data2.

Supplement: Figure 1—source data 2. [file elife-104580-fig1-data2.zip › Figure 1B-Source Data-2/Figure 1B_Source Data 2.pdf]

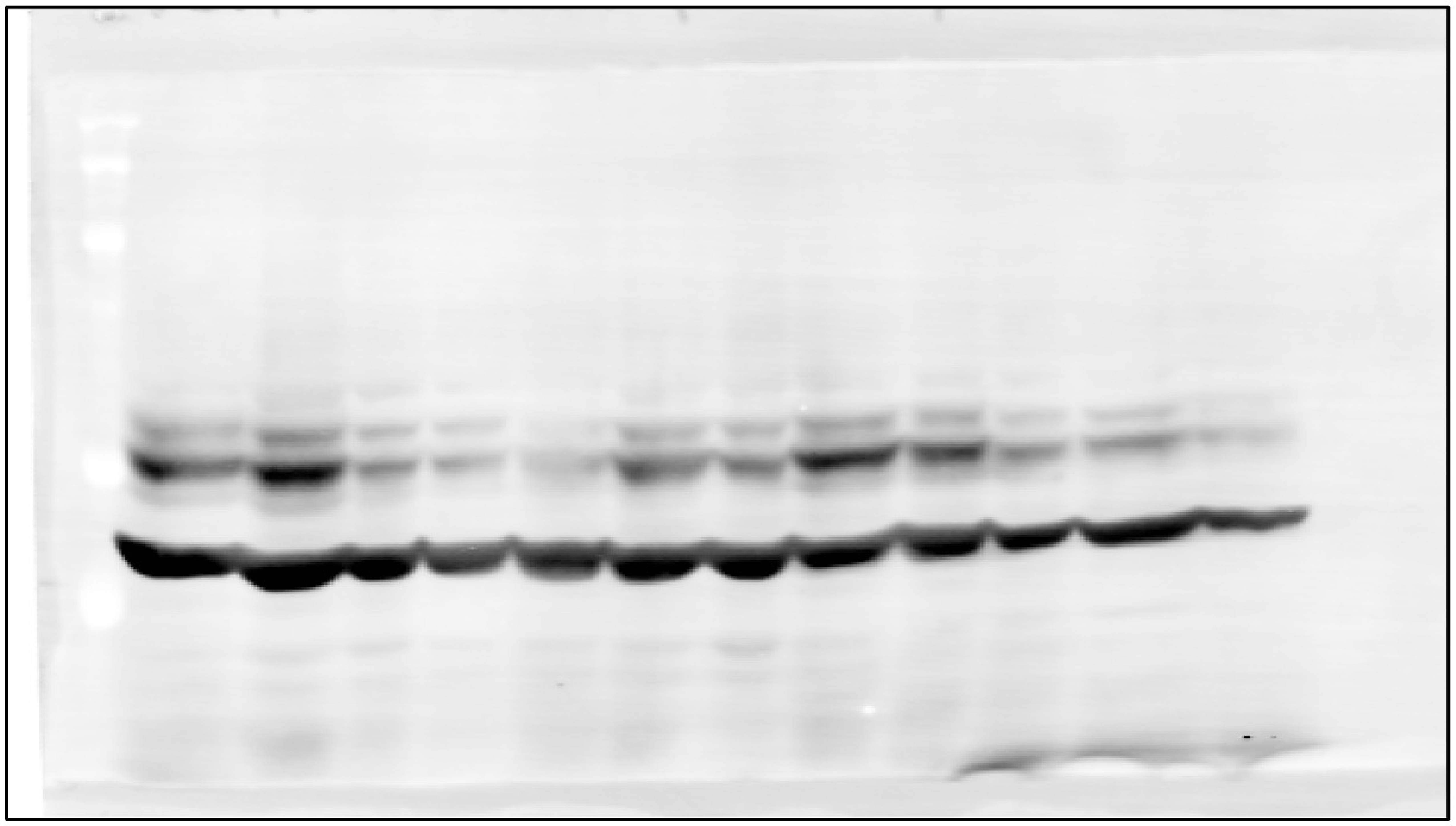

Supplement: Figure 2—source data 1. [file elife-104580-fig2-data1.zip › Figure 2B_Source Data-1/PexRAP cKO_R26-CreER_Actin.tif]

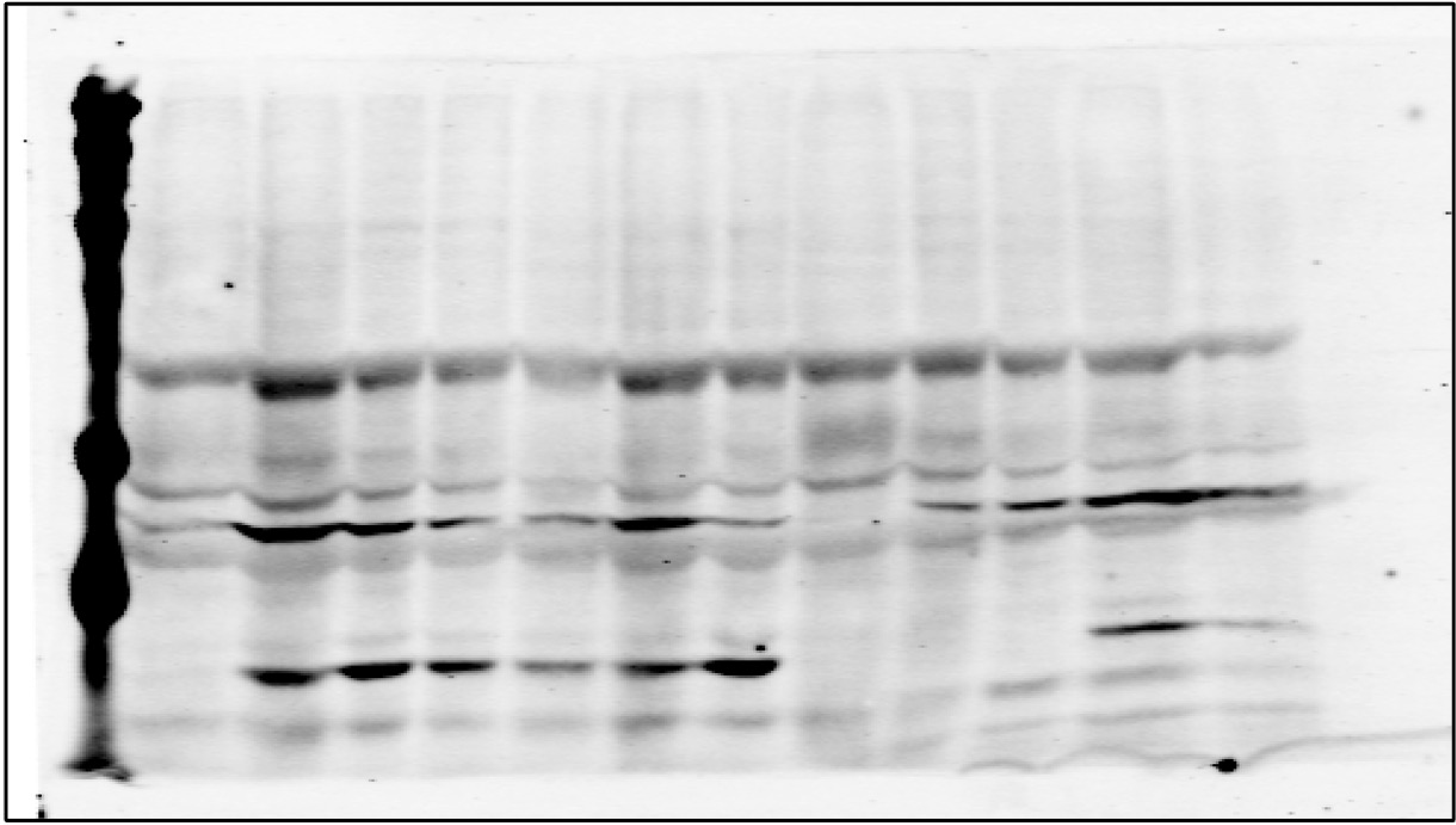

Supplement: Figure 2—source data 1. [file elife-104580-fig2-data1.zip › Figure 2B_Source Data-1/PexRAP cKO_R26-CreER_PexRAP.tif]

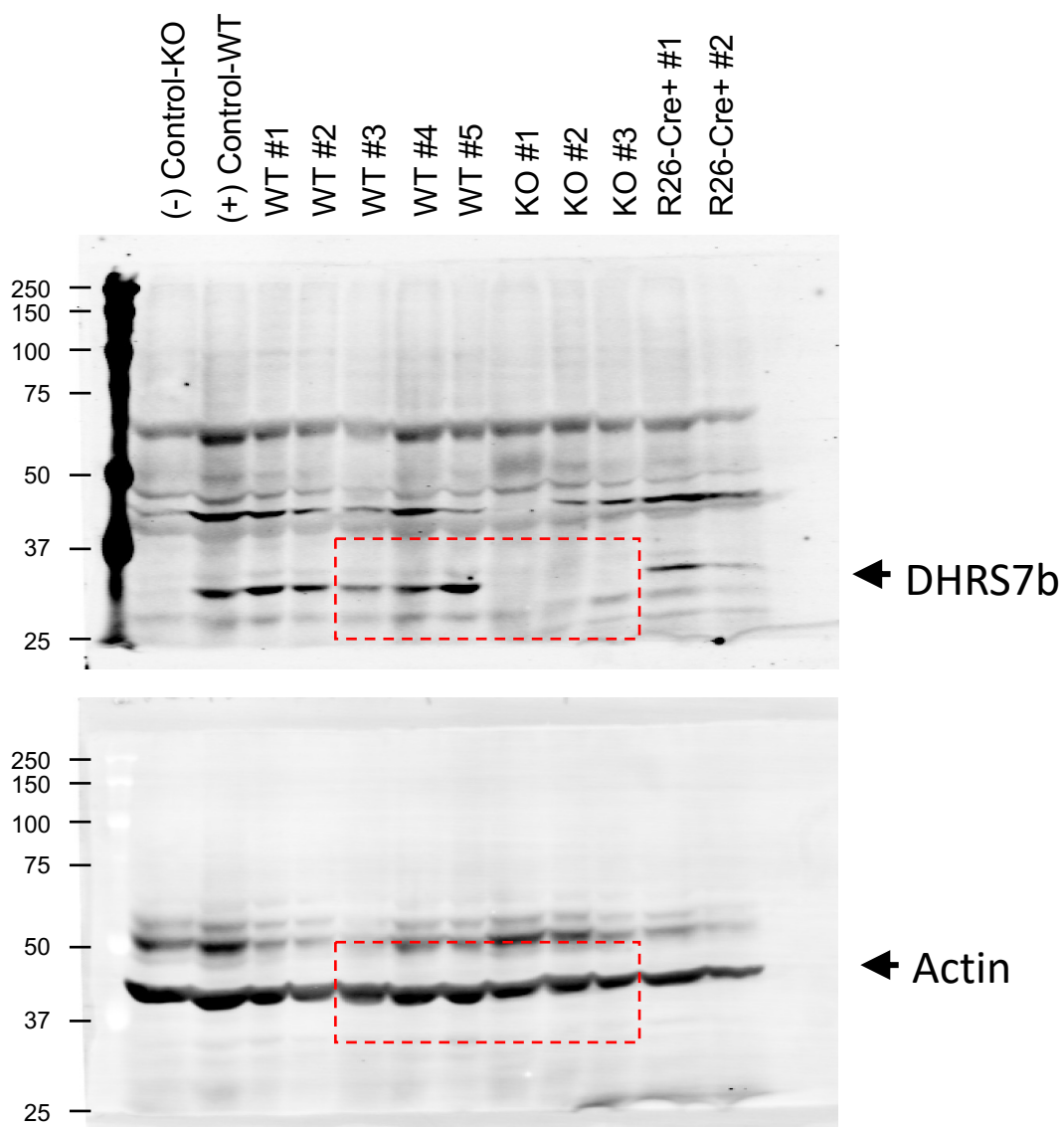

Figure 2B-Source Data2.

Supplement: Figure 2—source data 2. [file elife-104580-fig2-data2.zip › Figure 2B_Source Data-2/Figure 2B_Source Data-2.pdf]

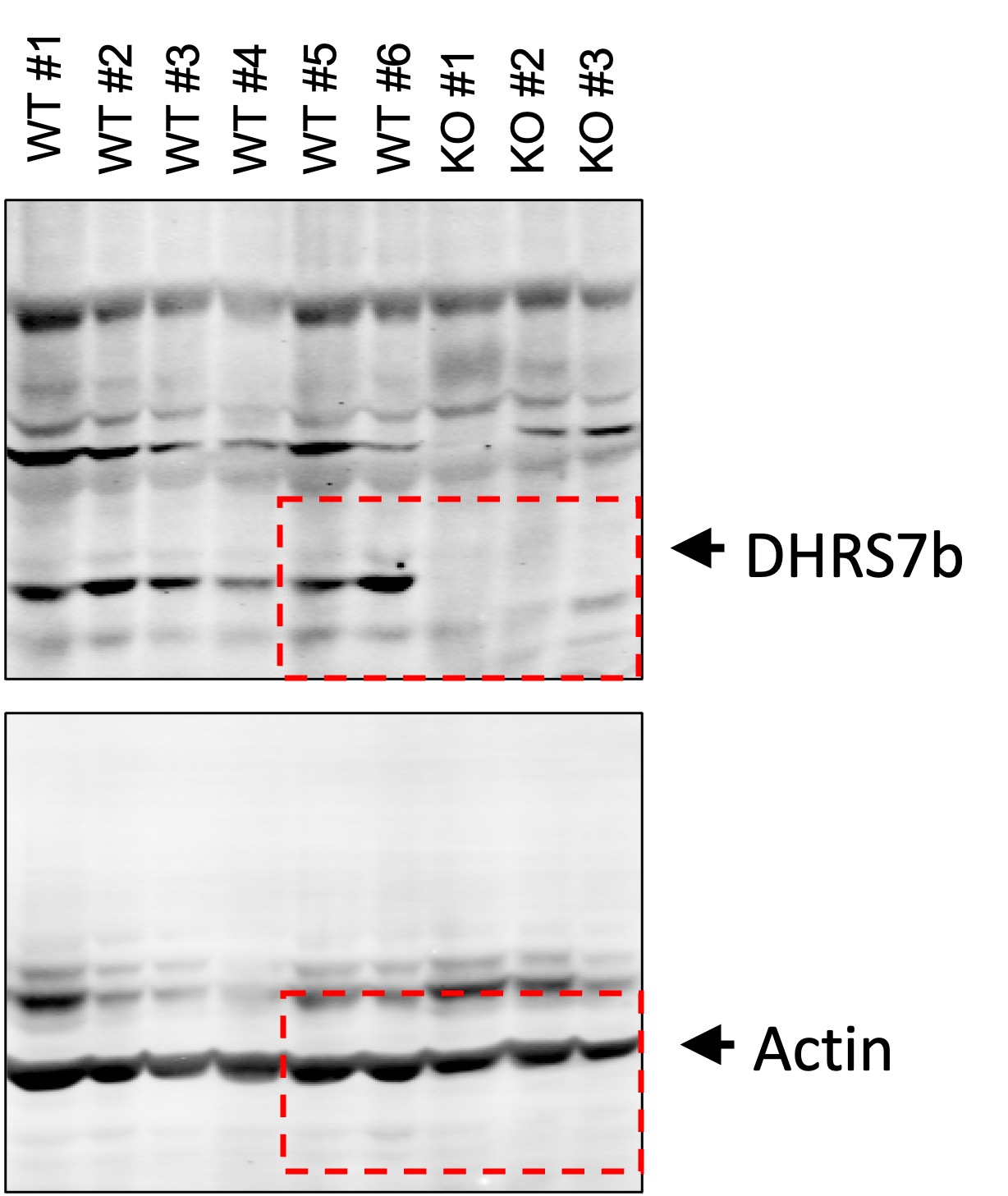

Supplement: Source data 1. [file elife-104580-data1.zip › Source Data/Figure 2-Source Data/Figure 2B.jpg]

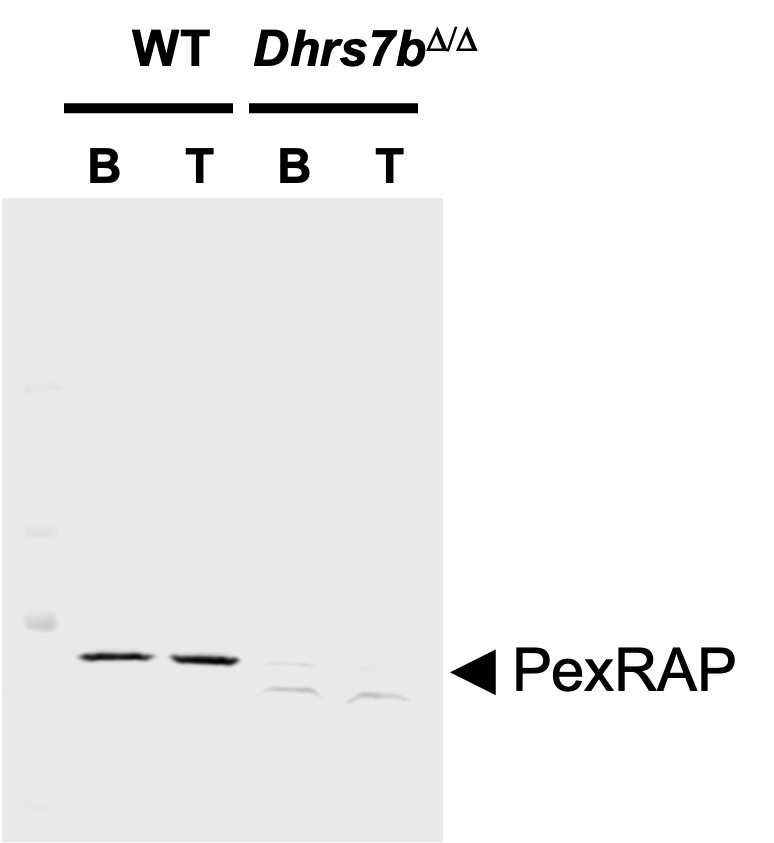

Supplement: Source data 1. [file elife-104580-data1.zip › Source Data/Figure 1-Source Data/Figure 1B_PexRAP.jpg]

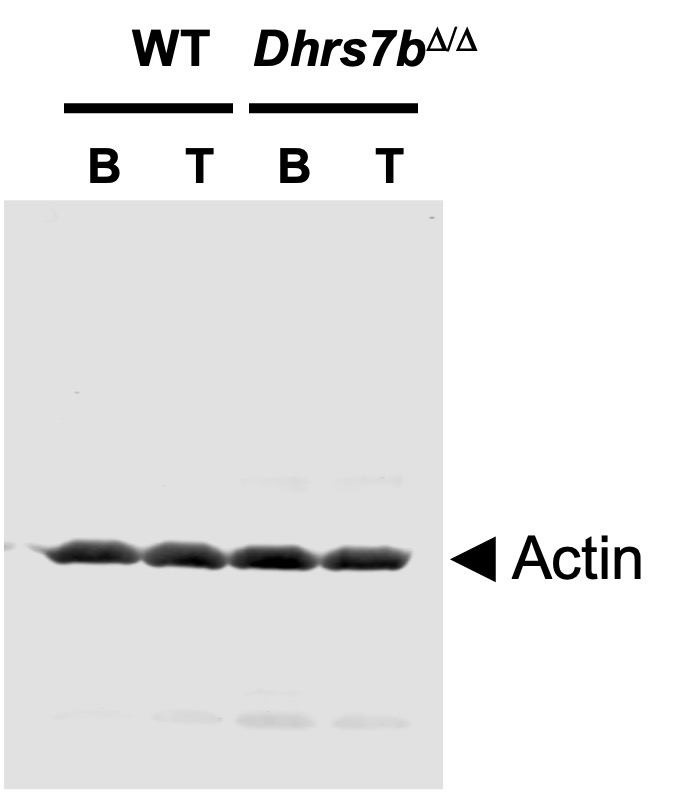

Supplement: Source data 1. [file elife-104580-data1.zip › Source Data/Figure 1-Source Data/Figure 1B_Actin.jpg]
